# Sinoatrial Nodal Artery Arising from the Right Posterolateral Artery: A Rare Anatomical Variant

**DOI:** 10.14797/mdcvj.1109

**Published:** 2022-09-06

**Authors:** Parviz-Ali Lotfian, Arun Umesh Mahtani, Seyed Zaidi, Richard Grodman

**Affiliations:** 1 Department of Medicine, Richmond University Medical Center/Mount Sinai, Staten Island, New York, US; 2 Department of Cardiology, SUNY Downstate Medical Center, Brooklyn, New York, US; 3 Department of Cardiology, Richmond University Medical Center/Mount Sinai, Staten Island, New York, US

**Keywords:** coronary vessels, anatomical variation, coronary circulation, NSTEMI

## Abstract

We discuss a case report of a 66-year-old male with no prior cardiac history who presented to the hospital with persistent hiccups and shortness of breath. Following a positive nuclear stress test and cardiac catheterization, a rare anatomical variant of a sinoatrial nodal artery originating from the right posterolateral artery was revealed.

A 66-year-old male with no prior cardiac history presented to the hospital with persistent hiccups, causing shortness of breath. A nuclear stress test showed a fixed perfusion defect involving the inferior wall, possibly due to diaphragmatic attenuation artifact with no evidence of stress-induced myocardial ischemia, and a mildly decreased left ventricular ejection fraction of 44% (Figure 1). Coronary angiography revealed two-vessel coronary artery disease, 80% stenosis of the middle right coronary artery (RCA), diffuse heavy calcification of the left anterior descending artery (LAD) involving the proximal and mid-portions with 80% maximal stenosis, a small caliber left marginal, and a sinoatrial (SA) nodal branch originating from the right posterolateral artery (RPLA) (Figure 2 A, B).

**Figure 1:**
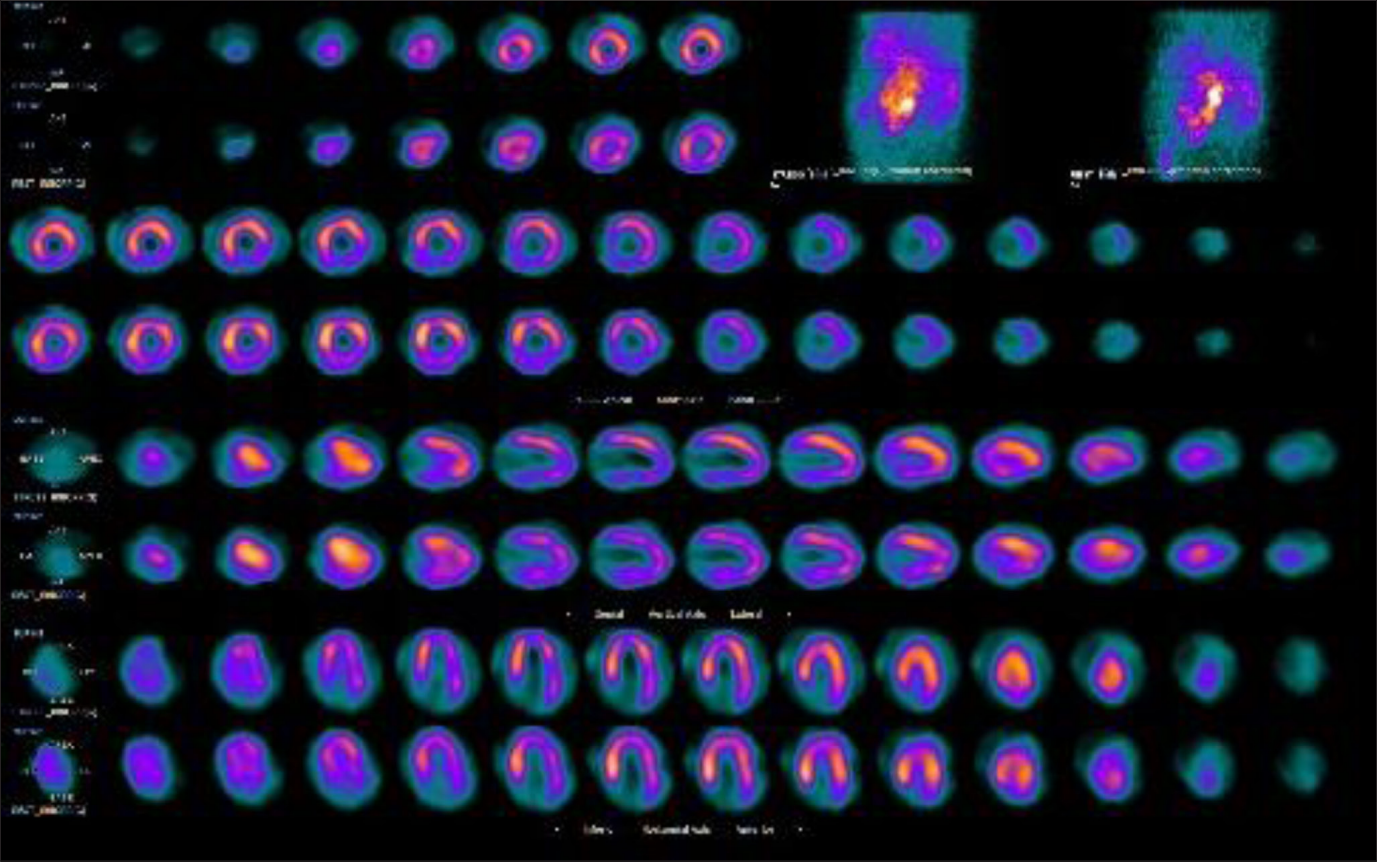
Nuclear stress test showing fixed perfusion defect in the inferior wall possibly due to diaphragmatic motion artifact.

**Figure 2:**
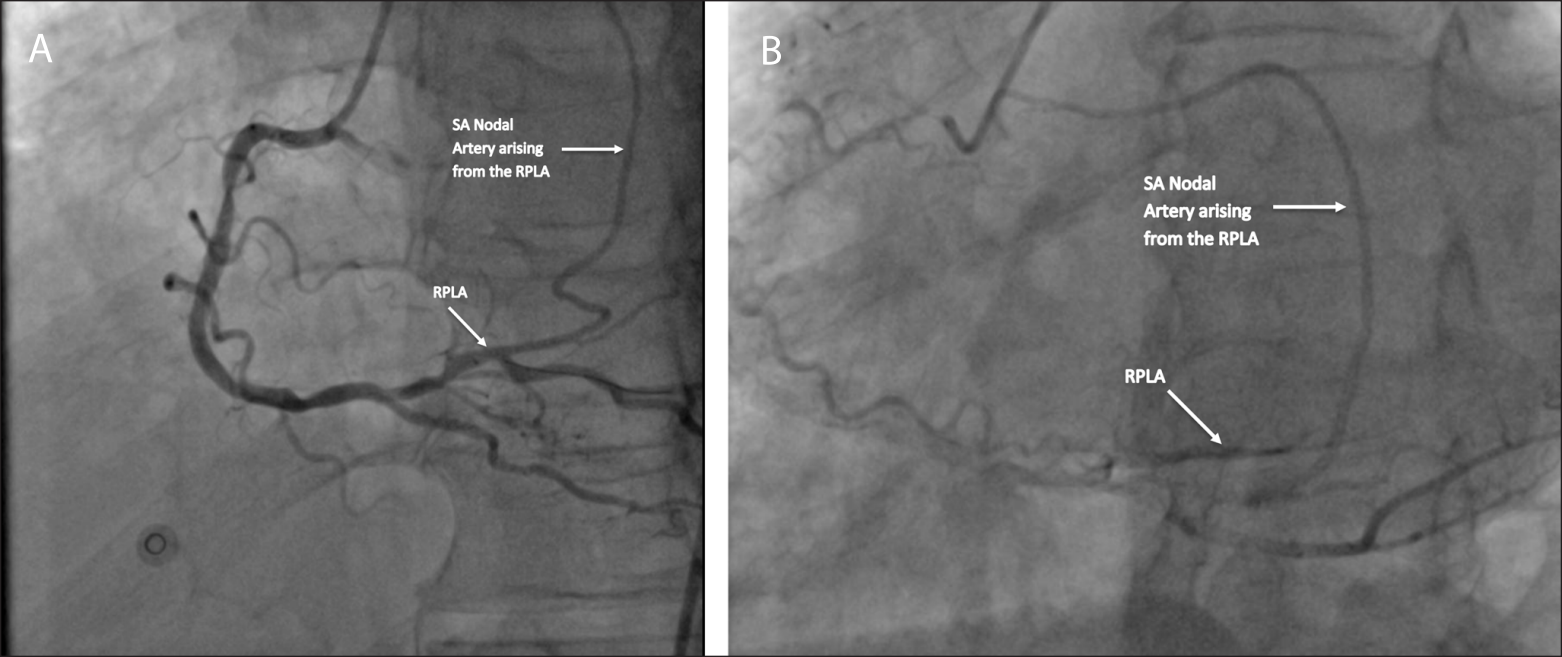
**(A, B)** Coronary artery angiography left anterior oblique view of the SA nodal artery arising from the right posterolateral artery. SA: sinoatrial

The SA nodal artery, a branch of the main coronary arteries, supplies blood to the SA node. The SA node is also known as the natural pacemaker of the heart. In 60% to 70% of cases, its blood supply originates from the RCA, and in 20% to 30% from the LAD and left circumflex coronary artery (LCX). The SA nodal artery provides vital oxygen and nutrients to the SA node, which is a key component in heart contraction that originates the initial electrical signal for atrial contraction.^1^ When originating from the RCA, the SA nodal artery most frequently arises at a mean distance of 1.2 cm (range 0.2–2.2 cm) from its beginning.^2^ In less than 1% of cases, the artery originates from the distal RCA.^3^ The posterolateral artery, also known as the posterior left ventricular artery, arises from the RCA in a typical dominant circulation. It is a terminal branch that supplies the inferior portion of the heart along with the posterior descending artery (PDA). It can also arise from the LAD or LCX.^4^ Based on available data, this the first documented case of an SA nodal artery originating from the RPLA.
